# Wavelength-Dependent Modulation of Mesenchymal Stem Cell Fate: A Systems Biology Framework for Tissue Repair and Regenerative Medicine

**DOI:** 10.3390/cells15100861

**Published:** 2026-05-08

**Authors:** Baptiste Amouroux, Kimia Motlagh Asghari, Morgane De Sousa, Virginie Gueguen, Cédric Chauvierre, Abolfazl Barzegari, Graciela Pavon-Djavid

**Affiliations:** 1 INSERM U1148, Laboratory for Vascular Translational Science, Nanotechnologies for Vascular Medicine and Imaging Team, Université Sorbonne Paris Nord, Université Paris-Cité, 99 Av. Jean-Baptiste Clément, 93430 Villetaneuse, France; baptiste.amouroux@univ-paris13.fr (B.A.); morgane.de-sousa@inserm.fr (M.D.S.); virginie.gueguen@univ-paris13.fr (V.G.); cedric.chauvierre@inserm.fr (C.C.); 2 Department of Medical Biotechnology, Faculty of Advanced Medical Sciences, Tabriz University of Medical Sciences, Tabriz 5165665931, Iran; 3 Research Center for Pharmaceutical Nanotechnology (RCPN), Tabriz University of Medical Sciences, Tabriz 5165665931, Iran

**Keywords:** mesenchymal stem cells (MSCs), photobiomodulation (PBM), dual photonic programming, secretome and paracrine signaling, redox biochemistry, dosimetry, light delivery, wavelength-specific signaling

## Abstract

**Highlights:**

**What are the main findings?**

- Red light (630–660 nm) and blue light (470–490 nm) activate orthogonal intracellular pathways in mesenchymal stem cells (MSCs), constituting a “Dual Photonic Program” in which red light drives mitochondrial anabolism while blue light primes the MSC secretome.
- Network meta-analysis (STRING/GO) identifies PI3K/Akt and RAB-GTPases as the central molecular hubs coordinating wavelength-specific regenerative responses in MSCs.

**What are the implications of the main findings?**

- In vitro optical filters (phenol red, polystyrene) systematically underestimate the effective delivered dose; applying the Arndt-Schultz biphasic law to corrected dosimetry resolves contradictory findings and defines the molecular thresholds between therapeutic priming and cellular exhaustion.
- This mechanistic framework provides a roadmap for transitioning from empirical photobiomodulation to standardized, mechanism-driven regenerative protocols tailored to the MSC’s anatomical origin.

**Abstract:**

Mesenchymal stem cells (MSCs) are central effectors in regenerative medicine, yet their clinical translation is hindered by inconsistent therapeutic outcomes and a lack of standardized light-delivery protocols. This review addresses an underexplored dimension of photobiomodulation (PBM): the divergent, wavelength-dependent signaling programs triggered in MSCs by red/near-infrared (NIR) versus blue light. By integrating biophysical principles of light delivery with a systems biology analysis of protein–protein interaction networks (STRING/GO), we delineate a “Dual Photonic Programming” framework. Red/NIR light (600–1100 nm) targets mitochondrial cytochrome c oxidase, activating a bioenergetic-anabolic program centered on PI3K/Akt/mTOR and Wnt/β-catenin—pathways essential for structural tissue repair. Blue light (400–500 nm) engages cytosolic flavins to drive a secretory-paracrine program that modulates vesicle trafficking and immunomodulatory cargo release. We further examine the dosimetric paradox, demonstrating how culture-environment optics and the Arndt–Schultz biphasic law govern the transition from regenerative stimulation to inhibitory oxidative stress. By tailoring photonic parameters to the MSC’s anatomical origin and metabolic baseline, PBM can serve as a high-fidelity bio-switch for orchestrated tissue repair, providing a mechanistic roadmap for standardized regenerative therapies.

## 1. Introduction

Mesenchymal stem cells (MSCs) have emerged as central effectors in regenerative medicine due to their capacity for multilineage differentiation, immunomodulation, and, more crucially, their sophisticated secretome [1,2]. This paracrine activity, encompassing a diverse array of cytokines, growth factors, and extracellular vesicles, plays a pivotal role in modulating inflammation, promoting angiogenesis, and orchestrating tissue repair. However, the translation of MSC-based therapies into consistent clinical outcomes is frequently hampered by the inherent variability in cellular responses to environmental cues. Consequently, developing non-invasive methods to precisely “prime” or steer MSC behavior is of paramount importance for the field.

MSCs are adult multipotent progenitors that can be isolated from a wide range of tissues, including bone marrow, adipose tissue, dental pulp, and umbilical cord, and share a minimal phenotypic identity: plastic adherence, co-expression of surface markers (CD73, CD90, and CD105), absence of hematopoietic markers (CD45^−^, CD34^−^, and HLA-DR^−^), and trilineage mesodermal differentiation capacity (osteogenesis, adipogenesis, and chondrogenesis) [3]. Beyond their differentiation potential, the therapeutic value of MSCs is increasingly attributed to their paracrine activity: a sophisticated secretome comprising cytokines, growth factors (TGF-β, VEGF, and IGF-1), and extracellular vesicles that orchestrate tissue repair, modulate inflammation, and promote angiogenesis [1,2,4].

A critical and underappreciated dimension of the phenotype of MSCs is their metabolic and redox heterogeneity. MSCs display phenotypic and metabolic heterogeneity dictated by their anatomical niche of origin [2]. This heterogeneity has direct consequences for PBM responsiveness. Bone marrow–derived MSCs (BM-MSCs), which display high mitochondrial mass and a strong osteogenic bias, preferentially respond to red/NIR light through cytochrome c oxidase (CCO) activation [5,6]. Adipose-derived MSCs (AD-MSCs) display a more pronounced glycolytic profile and a superior immunomodulatory secretome, with marked secretome remodeling in response to blue-light stimulation [7]. Dental pulp stem cells (DPSCs), which harbor a high flavin content, also respond to blue wavelengths with enhanced paracrine activity [8]. Umbilical cord–derived MSCs, known for their broad immunomodulatory profile, similarly show sensitivity to short-wavelength light [9]. This baseline variability extends to their endogenous antioxidant capacity and redox state, which fundamentally determines their sensitivity to external stimuli [10]. Consequently, the MSC phenotype is not a static entity but a dynamic state that can be selectively modulated by physical signals. This source-dependent photosensitivity implies that PBM protocols cannot be extrapolated across MSC types without characterizing the metabolic baseline of the target cell population. Photobiomodulation exploits this inherent plasticity, where different wavelengths act as bio-switches to initiate either mitochondrial anabolism or paracrine signaling, depending on the source-specific metabolism of the cell and its redox threshold [11].

Photobiomodulation (PBM), the application of non-ionizing electromagnetic energy to elicit biological responses, represents a powerful, label-free bioengineering tool for this purpose. By targeting endogenous chromophores, PBM can trigger specific signaling cascades that modulate metabolic flux and gene expression [10,11,12]. While the stimulatory effects of red and near-infrared (NIR) light on MSC proliferation and osteogenic differentiation are well-documented [11,13,14], the underlying molecular mechanisms often appear fragmented or inconsistent across different studies. Most studies examine isolated wavelengths, cell types, or readouts, resulting in an unclear and often contradictory picture of how light reprograms MSC biology. This lack of reproducibility is frequently attributed to the “black box” nature of cell–light interactions and a historical lack of dosimetric standardization.

A critical but overlooked dimension is that light is not a single stimulus. MSCs possess multiple endogenous photoreceptors, primarily mitochondrial cytochrome c oxidase for red/NIR wavelengths and flavins or cryptochromes for blue wavelengths, each initiating distinct photochemical reactions and divergent signaling trajectories [11,13,14]. Red illumination enhances mitochondrial respiration, generates controlled H_2_O_2_, and activates PI3K/Akt, ERK, and Wnt/β-catenin pathways, resulting in metabolic reinforcement and osteogenic programming [13,15,16]. In contrast, blue light produces cytosolic ROS microdomains, Ca^2+^ transients, and circadian-linked transcriptional responses, which amplify extracellular vesicle biogenesis and paracrine repair [17,18].

Despite these mechanistic differences, no conceptual framework has unified red and blue PBM into a coherent model of wavelength-dependent MSC programming. The absence of such a framework has limited the interpretation of experimental variability and hindered the rational design of PBM-based regenerative strategies. Most prior reviews address PBM as a general enhancer of cell function, implicitly assuming that wavelength effects are variations in magnitude rather than qualitatively distinct biological programs.

Here, we propose that MSCs operate under a dual photonic programming architecture, in which red and blue photons encode different biological information and selectively engage distinct intracellular networks. Through integration of mechanistic photoreception, ROS biochemistry, kinase signaling, calcium dynamics, and STRING/GO network analyses, we demonstrate that red PBM drives an anabolic, differentiation-oriented program, while blue PBM drives a secretory, paracrine-oriented program. These orthogonal signaling modes reflect the inherent bifurcation between mitochondrial and flavin/circadian photoreception, providing a mechanistic basis for the divergent outcomes reported across the PBM literature.

This conceptual framework extends beyond mechanistic organization and carries direct implications for regenerative medicine. Red PBM aligns with the metabolic and transcriptional requirements of bone and cartilage regeneration [19,20], whereas blue PBM enhances vascular repair, immunomodulation, and extracellular vesicle (EV)-based therapies [21,22]. The complementary nature of these programs further suggests the possibility, still unexplored experimentally, of designing sequential or combined multispectral PBM to generate multiphasic regenerative responses.

The clinical relevance of these signaling programs is directly tied to the target tissue context. Activation of PI3K/Akt/mTOR by red PBM reinforces the bioenergetic demands of osteoblast maturation, supporting bone defect repair and periodontal regeneration [19,20]. Concurrent Wnt/β-catenin activation promotes chondrogenic gene expression (SOX9, Type II collagen, and aggrecan), extending the benefit of red PBM to cartilage tissue engineering [23,24,25]. In contrast, the flavin-redox and Ca^2+^/MAPK cascade triggered by blue PBM amplifies EV biogenesis and cytokine release, addressing the paracrine requirements of wound healing, angiogenesis, and immunomodulation [18,21,22]. These pathway-tissue alignments provide the mechanistic rationale for wavelength-specific PBM protocols and are developed in detail in Section 5.1 and Section 5.2.

In this review, we propose a unifying conceptual framework: Dual Photonic Programming. We argue that PBM does not merely “stimulate” cells but rather executes specific biological programs dictated by the incident wavelength. We delineate two divergent signaling trajectories: (i) *Anabolic/Mitochondrial Programming*: Triggered by red light (600–660 nm), this program centers on the activation of cytochrome c oxidase (CCO). This photochemical event enhances mitochondrial respiration and ATP synthesis, generating controlled reactive oxygen species (ROS) microdomains that engage the PI3K/Akt/mTOR and Wnt/β-catenin pathways to favor cell survival and structural differentiation [13,15,16]. (ii) *Secretory/Paracrine Programming:* Induced by blue light (400–500 nm), this trajectory involves the photoexcitation of flavins and cryptochromes. This pathway facilitates a distinct redox signaling signature that reconfigures the MSC secretome, enhancing its immunomodulatory and chemoattractive properties [21,22].

Furthermore, we address the critical yet often overlooked impact of optical physics on biological outcomes. By integrating network-level protein analyses and redox biochemistry, we highlight how the divergence between nominal and effective irradiance, often caused by media absorption and heterogeneous beam profiles [26,27], shapes the biphasic dose-response of MSCs. By bridging the gap between photonic physics and cell biology, this review provides a comprehensive roadmap for the standardized application of PBM in advanced tissue engineering.

## 2. Physical Foundations of Photobiomodulation

### 2.1. Physical Determinants of Photon Delivery in Tissue and In Vitro Models

**Figure 1:**
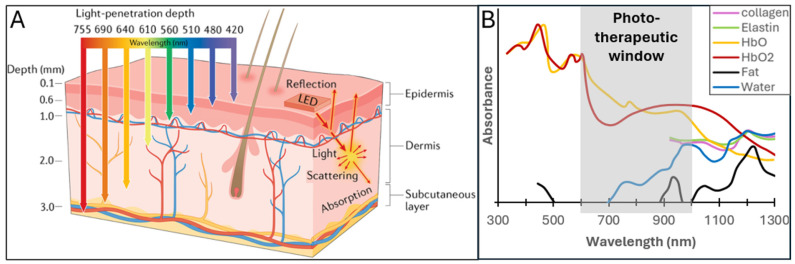
Light interaction with biological tissues and the phototherapeutic window relevant to photobiomodulation: (**A**) Schematic representation of wavelength-dependent light penetration and interaction with skin layers, illustrating reflection, scattering, absorption, and transmission. (**B**) Normalized absorption spectra of major tissue constituents, including water, hemoglobin (Hb and HbO_2_), collagen, elastin, and lipids, overlaid with the phototherapeutic window (gray). Panels adapted from Lee et al. [32] and Algorri et al. [31].

The therapeutic efficacy of PBM is fundamentally governed by the ability of photons to reach their intracellular targets at a specific irradiance. However, the path light travels from the source to the MSC is governed by complex optical laws that differ drastically between living tissue and laboratory environments. In biological tissues, light propagation is a complex process dominated by the competing phenomena of absorption and scattering (Figure 1A). Absorption is primarily dictated by endogenous chromophores, such as hemoglobin (both oxygenated and deoxygenated), melanin, and water, each possessing unique, wavelength-dependent extinction coefficients [28,29,30,31]. The widely recognized optical window (600–1100 nm) in the red and near-infrared (NIR) spectrum exists precisely because absorption from these primary pigments is minimized, allowing for deeper photon penetration into the dermal and sub-dermal layers where MSCs often reside.

In addition to absorption, scattering—due to differences in the refractive index between cellular components, membranes, and dense fibers of the extracellular matrix, such as collagen—disperses the light beam. This scattering reduces irradiance (mW/cm^2^) with depth, a factor that must be rigorously calculated when developing clinical protocols.

However, a significant translation gap occurs in in vitro modeling, where these tissue-level optics are often mistakenly assumed to be absent. In reality, the in vitro environment introduces its own set of complex physical barriers. The culture architecture acts as a multi-layered optical filter: the air–liquid interface causes initial reflection, while the culture medium itself, specifically when containing phenol red, exhibits significant absorption in the green and blue spectra. Furthermore, the polystyrene material of standard multi-well plates introduces a refractive-index mismatch that can cause beam divergence or internal reflection. Culture systems composed of hydrogels, decellularized matrices, or mineralized scaffolds exhibit refractive-index mismatches that can attenuate or redirect photons, thereby altering the effective intracellular dose, even under identical nominal irradiances [28,33,34].

As highlighted in the seminal study by Hadis et al. [16], these factors can lead to a significant discrepancy between the “nominal dose” (the energy emitted by the lamp) and the “effective dose” (the energy actually reaching the mesenchymal stem cell monolayer). Neglecting these variables results in inaccurate radiometric measurements, which remain a major cause of the lack of reproducibility and contradictory biological results observed in the literature on mesenchymal stem cell photobiomodulation (MSC-PBM). For a “dual photon programming” framework to be valid, it must be based on a precise quantification of the energy delivered at the cellular level.

These wavelength-dependent propagation differences determine not only how far photons travel but also which photoreceptors they encounter. Red and NIR photons reach the mitochondrial compartment, where components of the electron transport chain, including cytochrome c oxidase, absorb light and undergo redox modulation [15,16]. This leads to adjustments in mitochondrial membrane potential, ATP synthesis, and low-level ROS production, all of which contribute to lineage-specific signaling and metabolic adaptation. Blue wavelengths, in contrast, are absorbed predominantly by flavins and porphyrins, generating localized photochemical reactions that influence redox tone and calcium handling without substantial mitochondrial engagement [35,36,37]. The resulting distinction between deep-penetrating metabolic modulation and superficial oxidative signaling activation constitutes the mechanistic basis for the divergent MSC phenotypes elicited by red/NIR versus blue light.

Thus, the optical therapeutic window is not simply a descriptor of tissue transparency but the foundation upon which wavelength-specific photobiological mechanisms emerge in MSCs. Its constraints define the spatial domain of photon action, select the accessible chromophores, and ultimately dictate the qualitative nature of PBM-induced signaling. These physical determinants underpin all subsequent cellular responses and justify the mechanistic separation of PBM into red/NIR-driven metabolic pathways and blue-light–driven oxidative and secretory processes explored in later sections.

### 2.2. Endogenous Photoreceptors and Early Photophysical Events

Photobiomodulation in MSCs originates from the interaction of specific wavelengths with a limited set of endogenous chromophores whose spectral and biochemical properties determine the identity, localization, and kinetics of the earliest signaling events. In the red and near-infrared range, cytochrome c oxidase (CCO) is the most consistently implicated primary photoacceptor. Its heme-a, heme-a3, and copper centers display absorption bands overlapping the optical therapeutic window, and red illumination modulates the redox state and turnover rate of complex IV [11,13,14]. This photochemical modulation accelerates electron transfer, increases mitochondrial membrane potential, and elevates ATP synthesis, while simultaneously generating controlled mitochondrial ROS—primarily superoxide, which is rapidly dismutated to hydrogen peroxide—that function as signaling intermediates rather than toxic byproducts [15,38]. Red-light irradiation can also photodissociate nitric oxide from CCO, relieving NO-mediated inhibition of respiration and restoring oxidative phosphorylation under metabolic stress [13,15]. Together, these mechanisms position mitochondria as the central hub of red/NIR phototransduction, aligning photon absorption with metabolic gating, survival signaling, and lineage-specific programming in MSCs [16].

In contrast, blue-light photoreception is mediated by flavin-containing proteins and cryptochromes, whose chromophores (FAD and FMN) exhibit strong absorption peaks around 450 nm. Cryptochromes (CRY1 and CRY2) are flavoproteins whose FAD-dependent absorption spectra peak in the blue-light range, providing a photophysical basis for wavelength-specific photobiomodulation and its coupling to circadian and transcriptional regulation [39].

**Figure 2:**
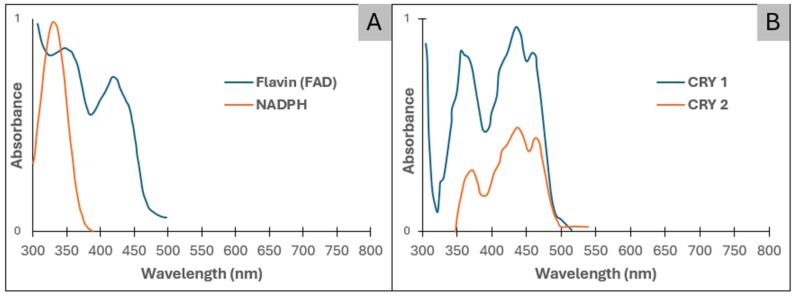
Absorption spectra of endogenous photoreceptors involved in blue-light photobiomodulation: (**A**) Absorption spectra of flavins (FAD) and flavin-bound NADPH-dependent enzymes, highlighting strong absorption in the blue-light range relevant to photobiomodulation. Adapted from Ernst et al. [25]. (**B**) Absorption spectra of FAD-containing cryptochromes CRY1 and CRY2, supporting their role as blue-light photoreceptors linking photonic input to circadian and transcriptional regulation. Adapted from Vanhaelewyn et al. [39].

The strong absorption of blue wavelengths by flavin-based chromophores and cryptochromes is illustrated by their characteristic absorption spectra (Figure 2), providing a photophysical basis for wavelength-specific blue-light photobiomodulation. Upon excitation, flavins undergo ultrafast electron-transfer reactions that produce spatially confined ROS pulses, including superoxide, hydrogen peroxide, singlet oxygen, and semiquinone radicals, whose lifetimes and diffusion properties differ markedly from mitochondrial ROS [36,40,41]. These short-lived oxidative microdomains modulate cysteine-based redox switches on kinases, phosphatases, and ion channels, thereby reorganizing signaling networks without inducing generalized oxidative stress [17,41]. Cryptochromes constitute an additional blue-light sensor class: as flavoproteins undergoing photo-reduction cycles, they integrate photonic input into circadian transcriptional machinery through interactions with CLOCK/BMAL1 complexes [37,42,43,44]. Blue-light PBM, therefore, links photochemistry to transcriptional reprogramming, metabolic oscillations, and stress-response gene expression, establishing a mechanistic route unavailable to red/NIR wavelengths.

A further distinguishing feature of blue-light signaling is its capacity to remodel calcium dynamics. ROS generated by flavin excitation can modulate redox-sensitive Ca^2+^ channels or pumps, producing transient Ca^2+^ elevations that propagate signals to the cytoskeleton, focal adhesions, and vesicular trafficking machinery [17]. Because MSC extracellular vesicle biogenesis is tightly coupled to redox and calcium fluctuations, these early photochemical events provide a direct explanation for the enhanced EV release and altered cargo composition consistently observed under blue-LED irradiation [18,45]. These effects occur independently of mitochondrial engagement and, therefore, do not induce the bioenergetic reinforcement characteristic of red PBM; rather, blue light establishes a secretory and communication-oriented phenotype that aligns with MSC paracrine function.

Taken together, these wavelength-dependent phototransduction mechanisms create two coherent but mechanistically distinct modes of PBM action. Red and NIR wavelengths engage mitochondrial photoacceptors to reprogram bioenergetics, antioxidant signaling, and anabolic activity, whereas blue light activates flavin- and cryptochrome-dependent photochemistry to generate oxidative microdomains, calcium remodeling, and circadian-linked transcriptional shifts. The divergence of these early events reflects not simply differences in spectral absorption but the fundamentally distinct intracellular landscapes of photon accessibility and biochemical reactivity. This mechanistic partition underlies the wavelength-specific MSC behaviors explored in the next sections.

### 2.3. Why MSCs Are Uniquely Photomodulable

Mesenchymal stem cells occupy a metabolic and signaling landscape that makes them exceptionally responsive to photobiomodulation. Unlike terminally differentiated cells, MSCs sustain a highly plastic bioenergetic profile in which glycolysis, oxidative phosphorylation, mitochondrial dynamics, and redox buffering operate in a finely regulated equilibrium that shifts during proliferation, commitment, and matrix synthesis [3,5]. This intrinsic plasticity creates multiple biochemical entry points through which light-dependent processes can exert a disproportionate influence on cell fate [46,47].

A defining feature of MSC biology is the tight coupling between mitochondrial performance and lineage specification. Osteogenic and chondrogenic differentiation require increases in oxidative phosphorylation, expansion of mitochondrial mass, remodeling of cristae, and restructuring of the TCA cycle [3,48]. Even modest adjustments in mitochondrial membrane potential or ATP availability can redirect commitment trajectories [49,50]. Red and near-infrared PBM interact directly with this metabolic circuitry by modulating cytochrome c oxidase activity, enhancing respiratory flux, and generating controlled mitochondrial ROS signals that engage PI3K/Akt, ERK1/2, Nrf2, and Wnt/β-catenin pathways [19,51]. Because these pathways constitute core regulators of MSC survival and differentiation, photonic modulation of their upstream metabolic nodes produces amplified biological outputs disproportionate to the physical stimulus [52].

The redox environment of MSCs further enhances their photomodulation capability. MSCs maintain relatively low basal ROS levels yet rely on transient oxidative pulses to regulate self-renewal, proliferation, and differentiation [53,54]. Such reliance on redox-sensitive checkpoints renders them receptive to both mitochondrial H_2_O_2_ signals elicited by red/NIR PBM and to cytosolic ROS microdomains generated by flavin-based blue-light photochemistry [13,55]. These ROS signatures integrate with antioxidant networks and the Nrf2-Keap1 axis, shaping stress tolerance, secretory profiles, and regenerative potential. Cells whose function is intrinsically regulated by finely tuned redox gradients are, therefore, naturally susceptible to photonic cues that modulate these gradients with spatial and kinetic specificity [4].

Beyond metabolism and redox control, MSCs display a secretome architecture that is highly sensitive to intracellular Ca^2+^, cytoskeletal tension, and vesicular trafficking [56]. Flavin-mediated ROS pulses and cryptochrome-associated signaling alter cytosolic redox status, calcium handling, and circadian-linked transcriptional programs, thereby reprogramming extracellular vesicle release and cargo loading under blue-light PBM [57,58,59]. Because MSC therapeutic activity in vivo is largely mediated by their paracrine function rather than long-term engraftment, even modest shifts in secretory behavior can translate into substantial functional differences at the tissue level [60,61]. Blue-light PBM thus interfaces naturally with the major mechanism through which MSCs exert regenerative effects.

Finally, the vulnerability of MSCs to pathological microenvironments further accentuates their apparent sensitivity to PBM. In conditions such as diabetes, hypoxia, chronic inflammation, or oxidative stress, mitochondrial function and redox homeostasis are compromised, reducing reparative efficacy [58]. PBM, particularly in the red/NIR range, can restore mitochondrial competence, decrease apoptosis, and re-establish secretory profiles compatible with tissue repair [62,63]. The same photophysical mechanisms that yield modest enhancements in unstressed MSCs can, therefore, generate pronounced benefits under pathological conditions, amplifying the overall photobiological response.

Collectively, MSC photomodulation capability does not arise from an intrinsic optical sensitivity but from the convergence of three major cellular properties: a highly plastic metabolic network controlled by mitochondrial checkpoints [5,64,65]; a redox-regulated signaling architecture tuned to low-amplitude oxidative fluctuations [4,66]; and a secretory apparatus tightly coupled to ROS, Ca^2+^, and circadian control [2,56]. PBM is mechanistically aligned with each of these layers, allowing photons to act as efficient regulators of MSC fate and function despite minimal energy input. This alignment underpins the wavelength-specific behaviors and explains why MSCs consistently emerge as one of the most photobiomodulable cell types in regenerative medicine [61,67].

### 2.4. Light Sources and Dosimetry

The interpretation of PBM outcomes in MSCs requires careful consideration of the illumination source and the quantitative features of light delivery. Although laser diodes and LEDs differ fundamentally in beam coherence and collimation, these optical distinctions lose biological relevance once photons enter scattering tissue-like media, where coherence decays rapidly. What ultimately governs the magnitude and quality of MSC responses is the *effective photon dose* delivered to intracellular targets, a parameter shaped by irradiance, fluence, spectral bandwidth, and exposure geometry rather than by intrinsic properties of the light source itself [68,69].

LEDs have become widely adopted in MSC photobiology because they enable homogeneous illumination across large culture surfaces and 3D constructs with minimal thermal perturbation. Laser systems, conversely, provide high radiance and well-defined beam profiles but require precise control to avoid local overexposure and steep energy gradients, particularly when irradiation occurs through optically heterogeneous matrices [70]. As several studies have shown, the nominal irradiance measured at the device output often diverges substantially from the photon density that actually reaches cells due to cumulative absorption, scattering, and reflection within the culture medium and scaffolding materials [70]. This divergence has contributed to the variability in the PBM literature and underscores the need for rigorous dosimetric calibration. Similar discrepancies between incident and effective photon flux have been quantified in the context of upconverting nanoparticle “nanolamps”, where actinometric measurements revealed that only a fraction of the NIR excitation is converted into usable visible photons, despite apparently high input power [71]. An overview of photobiomodulation parameters reported across stem cell studies, including wavelength ranges, irradiance, fluence, and exposure duration, is summarized in Table 1, based on a large-scale literature compilation and current recommendations from the World Association for Photobiomodulation Therapy (WALT) [10].

**Figure 3:**
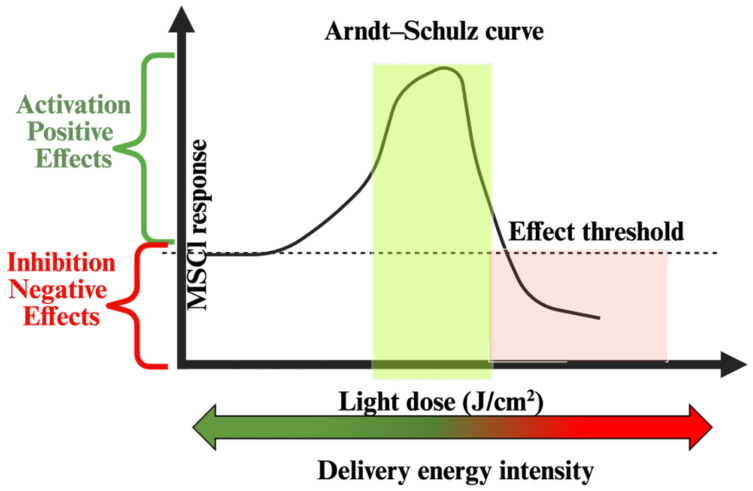
Biphasic dose-response behavior in photobiomodulation (Arndt–Schulz principle). Schematic representation of the biphasic relationship between light dose and biological responses in photobiomodulation. Low-to-intermediate doses elicit stimulatory effects, whereas higher doses progressively lead to inhibitory or detrimental responses. The highlighted region indicates a wavelength- and context-dependent effective dose range, within which photobiomodulation is most likely to support beneficial cellular signaling outcomes. This conceptual framework applies to multiple light-induced processes, including redox signaling, metabolic modulation, and calcium-dependent pathways.

Beyond absolute energy delivery, the spatiotemporal structure of illumination governs whether PBM elicits stimulatory, neutral, or inhibitory effects. This non-linear dose-response behavior is classically described by the Arndt–Schulz principle, which provides a conceptual framework for interpreting both stimulatory and inhibitory effects of photobiomodulation depending on the delivered light dose (Figure 3). MSCs, like other photobiological systems, exhibit a characteristic biphasic response in which subthreshold fluences fail to trigger signaling, intermediate doses enhance mitochondrial and redox pathways, and higher exposures converge toward metabolic inhibition. This non-linearity is particularly evident when comparing blue and red wavelengths: blue photons, being absorbed more superficially, require lower fluences to induce ROS- or calcium-driven signaling events, whereas red/NIR wavelengths demand higher doses to ensure sufficient penetration and interaction with mitochondrial chromophores [27].

**Figure 4:**
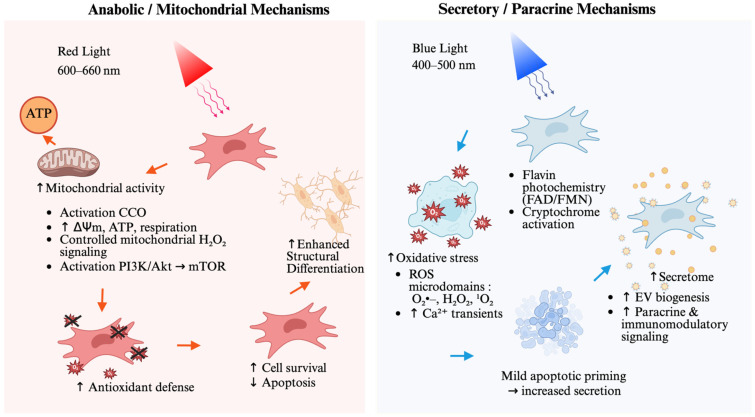
Dual photonic programming of mesenchymal stem cell fate and secretory function. This schematic illustrates the wavelength-dependent bifurcation of intracellular signaling architectures and functional bias in mesenchymal stem cells (MSCs) under photobiomodulation: Left: Anabolic/mitochondrial-associated programming (red and near-infrared light, 600–660 nm). Red and near-infrared wavelengths predominantly engage mitochondrial chromophores, including cytochrome c oxidase (CCO), increasing mitochondrial membrane potential (ΔΨm), ATP synthesis, and respiratory chain activity. Controlled production of mitochondrial hydrogen peroxide (H_2_O_2_) acts as a redox signaling intermediate that activates PI3K/Akt/mTOR and Wnt/β-catenin pathways. This signaling architecture reinforces antioxidant defenses, promotes cell survival, suppresses apoptosis, and favors differentiation-compatible, matrix-producing functional states associated with osteogenic and structurally regenerative outcomes. Right: Secretory/paracrine programming (blue light, 400–500 nm). Blue wavelengths are absorbed by cytosolic flavin-containing molecules (FAD/FMN) and cryptochromes, triggering photochemical reactions that generate spatially confined cytosolic ROS microdomains (O_2_•−, H_2_O_2_, ^1^O_2_) and Ca^2+^ transients. These redox and ionic signals converge to activate secretory and adaptive programs, driving enhanced extracellular vesicle (EV) biogenesis and amplified paracrine and immunomodulatory signaling toward target tissues. Under specific dosimetric conditions, mild apoptotic priming below the cell-death threshold further amplifies secretome release and microenvironmental remodeling, collectively biasing MSCs toward a communication-oriented, high-paracrine-potency functional state.

Standardization of reporting parameters, therefore, remains essential. Variations in beam size, working distance, spectral composition, and sample holder geometry can alter the intracellular photon budget by orders of magnitude, even when reported irradiance values appear identical between studies. Without explicit characterization of these optical factors, comparative interpretation of PBM effects on MSC proliferation, differentiation, and secretory activity becomes unreliable. A mechanistic understanding of MSC photobiology thus requires integrating not only wavelength-dependent tissue optics but also the physical determinants of photon delivery that condition all downstream signaling events. To integrate these photophysical and dosimetric constraints with wavelength-dependent cellular responses, Figure 4 provides a schematic overview of the proposed dual photonic programming framework, illustrating how red/near-infrared and blue wavelengths bias mesenchymal stem cell functional states through distinct intracellular signaling architectures.

## 3. Wavelength-Specific Mechanisms in MSCs

Photobiomodulation elicits wavelength-dependent signaling programs in MSCs that originate from fundamentally distinct photoreceptors and photochemical reactions. Red and near-infrared wavelengths engage mitochondrial chromophores to modulate bioenergetics and anabolic signaling, whereas blue light interacts with flavin- and porphyrin-based systems to generate oxidative microdomains, calcium remodeling, and transcriptional shifts. These divergent early events propagate through separate intracellular networks and produce functionally distinct MSC phenotypes relevant for regenerative medicine.

### 3.1. Red-Light Signaling: Mitochondrial Phototransduction, Metabolic Reinforcement, and Anabolic Programming

Red and near-infrared wavelengths initiate a phototransduction cascade centered on cytochrome c oxidase (CCO), whose heme and copper centers absorb within the optical therapeutic window [58]. Photon absorption increases CCO turnover, accelerates electron transport, and elevates mitochondrial membrane potential, generating a modest rise in mitochondrial superoxide that is rapidly converted into hydrogen peroxide [13,15]. This H_2_O_2_ signal constitutes the principal redox mediator through which red PBM modulates kinase activity, phosphatase inhibition, and transcription factor activation.

Akt/mTOR signaling is among the first pathways influenced by red PBM, reflecting the coupling between ATP availability, nutrient sensing, and protein synthesis. Enhanced oxidative phosphorylation stimulates mTORC1 through Rheb-dependent activation, thereby reinforcing anabolic metabolism and promoting differentiation [51,72]. Concurrently, ERK1/2 activation responds to H_2_O_2_-mediated modulation of upstream redox-sensitive phosphatases, supporting early proliferation and survival [72]. These pathways converge with Wnt/β-catenin signaling, a central driver of osteogenesis, by integrating redox signals into β-catenin stabilization and nuclear translocation [48,73].

The anabolic phenotype characteristic of red-light PBM is evidenced by increased expression of osteogenic markers, including Runx2, Osterix, alkaline phosphatase, and Type I collagen [74,75,76]. Red PBM also mitigates apoptosis and improves mitochondrial fitness under inflammatory, diabetic, or hypoxic stress [16,47,77], restoring the reparative potential of compromised MSCs. Notably, red-light illumination promotes mitochondrial biogenesis, cristae restructuring, and overall respiratory competence [13,38], features that enable MSCs to undertake energy-demanding processes such as matrix synthesis and mineralization.

Collectively, red PBM acts as a metabolic amplifier, reinforcing survival pathways, enhancing bioenergetic efficiency, and promoting differentiation through mitochondrial control points. Its impact reflects not only photonic stimulation but the intrinsic dependency of MSC fate on mitochondrial performance and redox homeostasis.

### 3.2. Blue-Light Signaling: Flavin Photochemistry, Oxidative Microdomains, Ca^2+^ Transients, and Secretory Programming

Blue-light photobiology originates from the excitation of flavoproteins and porphyrin-like chromophores whose absorption maxima lie near 450 nm. Upon illumination, FAD/FMN cofactors undergo ultrafast photoreduction, generating superoxide, hydrogen peroxide, singlet oxygen, and semiquinone radicals [36,78]. These ROS are produced in spatially restricted cytosolic microdomains rather than in mitochondria, giving blue PBM a distinctive oxidative signature with unique downstream consequences [17,79].

These oxidative microdomains activate redox-sensitive pathways such as NRF2, p38 MAPK, and JNK, reshaping transcriptional programs linked to stress adaptation and paracrine communication [58]. In parallel, cryptochrome photoreduction introduces a wavelength-specific transcriptional layer: CRY1/CRY2 undergo conformational changes that modulate interactions with CLOCK/BMAL1 complexes, linking blue illumination to circadian control of metabolic and secretory genes [44,80]. This mechanism is exclusive to blue light and has no analog in red/NIR PBM.

Importantly, core circadian clock components, including CRY1, CRY2, BMAL1, and PER2, are functionally expressed in human bone marrow– and adipose-derived MSCs, where they actively regulate differentiation, migration, and cell cycle progression [81]. In MSCs, CRY2 suppresses RUNX2-dependent osteogenic programs through inhibition of the BMAL1–CLOCK/P300 complex, while CRY1 modulates adipogenesis via Wnt/β-catenin signaling [82]. These data establish that the circadian machinery is not merely present in MSCs but actively gates their fate decisions, providing a direct mechanistic basis through which blue-light photoreduction of cryptochromes can influence lineage commitment and secretory programming in a cell-type–specific manner.

A defining feature of MSC responses to blue PBM is the remodeling of intracellular calcium. ROS microdomains modulate redox-sensitive Ca^2+^ channels and pumps, triggering transient increases in cytosolic Ca^2+^ that propagate signals to the cytoskeleton, focal adhesions, and vesicular trafficking machinery [55,83]. This provides a mechanistic foundation for the well-documented increase in extracellular vesicle (EV) release and alterations in EV cargo observed in blue-irradiated MSCs [17,83]. The EVs produced under blue PBM exhibit enhanced pro-angiogenic and immunomodulatory activities, reflecting the tight integration between redox/circadian cues and secretion biology.

At the subcellular level, the oxidative microdomains generated by flavin photochemistry may engage a sublethal caspase-3 activation cascade recently identified as a key regulator of apoptotic exosome-like vesicle (ApoExo) biogenesis. Beillevaire et al. demonstrated that caspase-3 controls the fusion of autolysosomes, formed by convergence of multivesicular bodies and autophagosomes, with the plasma membrane, enabling EV release independently of frank cell death [84].

In this framework, blue-light ROS pulses would not drive apoptosis but prime a sublethal caspase pathway that amplifies vesicular trafficking. This mechanism is consistent with non-apoptotic roles of caspase-3 in stem cell biology, where sublethal caspase activation promotes differentiation and paracrine activity [85] and provides a molecular explanation for the enhanced EV biogenesis and immunomodulatory cargo alterations observed under blue-LED irradiation of MSCs.

Functionally, blue PBM favors a secretory phenotype, enhancing paracrine activity rather than promoting anabolic differentiation. The distinction arises from the nature of the photoreceptors and ROS involved: blue light does not reach mitochondria efficiently and, therefore, does not activate the bioenergetic reinforcement required for osteogenic programming. Instead, it modulates cytosolic signaling circuits that regulate communication and stress adaptation.

### 3.3. Dose-Dependence, Temporal Dynamics, and Context Sensitivity

The biphasic nature of PBM imposes stringent dose constraints: insufficient fluence yields no measurable effect, whereas excessive exposure, especially in the blue range, induces oxidative stress and decreased viability [17,46,79]. Moreover, the temporal structure of illumination (continuous vs. pulsed) shapes ROS kinetics, mitochondrial responses, and EV secretion patterns. Contextual factors such as hypoxia, inflammation, aging, or diabetes further modulate PBM responses by altering mitochondrial resilience, antioxidant capacity, and secretory activity [47,86,87,88]. These dependencies highlight the need for mechanistically informed dosing strategies tailored to the biological objective: metabolic reinforcement (red PBM) or secretory enhancement (blue PBM).

## 4. Network-Level Interpretation of Photonic Responses in MSCs

Gene sets for network analysis were curated from experimental studies identified during the literature review. Proteins were selected on the basis of consistent experimental evidence linking their expression, activity, or regulation to red/NIR light photobiomodulation (n = 36 proteins) or blue-light photobiomodulation (n = 57 proteins) in mesenchymal stem cells or closely related primary cell types. The complete protein lists for each wavelength condition are provided in Supplementary Table S1.

Protein–protein interaction (PPI) networks were independently constructed for each gene set using the STRING database (version 12.0; https://string-db.org; accessed on 5 January 2026), with Homo sapiens as the reference organism and a minimum required interaction score of 0.700 (high confidence). This threshold restricts the analysis to interactions supported by robust experimental or curated database evidence. Networks were imported into Cytoscape (version 3.x) for visual refinement: node color coding was applied manually to highlight functionally related protein clusters on the basis of Gene Ontology biological process annotations. Final network figures were exported in PDF format.

Network topology statistics were computed within STRING. For the blue-light network (55 nodes, 235 edges; expected edges: 22; average node degree: 9.04; average local clustering coefficient: 0.653), the PPI enrichment *p*-value was <1.0 × 10^−16^, confirming that the selected proteins form a biologically coherent interaction module rather than a random assembly. Equivalent network statistics were computed for the red/NIR network (35 nodes).

Gene Ontology (GO) biological process enrichment analysis was performed using the functional enrichment tool integrated within STRING v12.0, applied to the same protein lists. Statistical significance was assessed against a whole-genome background and adjusted for multiple testing using the Benjamini–Hochberg false discovery rate (FDR) procedure; terms with FDR < 0.05 were retained. Results were visualized as bubble plots ranked by −log_10_(FDR), with semantically related terms grouped at a similarity threshold ≥ 0.8. The ten most significant non-redundant GO biological process terms are reported for each wavelength condition in Supplementary Figure S1.

**Figure 5:**
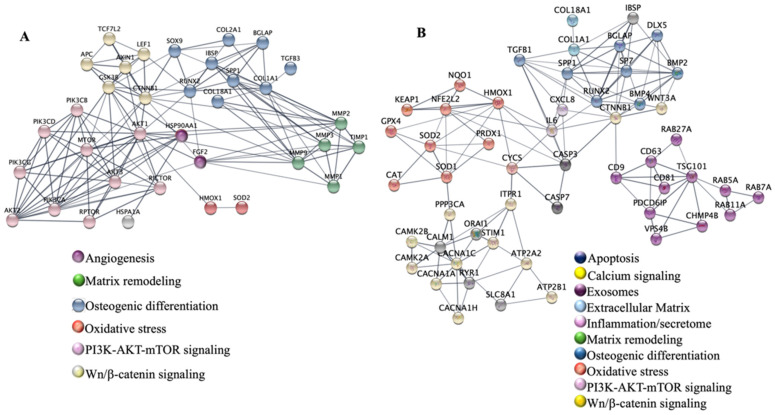
Network-level functional organization of red-light– and blue-light–responsive genes in mesenchymal stem cells. Protein–protein interaction (PPI) networks were generated using the STRING database and visualized in Cytoscape for gene sets reported in the literature as responsive to red-light (**A**) or blue-light (**B**) photobiomodulation in MSCs. Nodes represent proteins and edges represent known or predicted interactions. Node colors correspond to dominant functional annotations assigned based on Gene Ontology and pathway enrichment analyses combined with the literature curation. For red-light–responsive genes (**A**), the network is primarily organized around modules associated with osteogenic differentiation (blue), PI3K–AKT–mTOR signaling (violet), oxidative stress and redox regulation (red), matrix remodeling (green), Wnt/β-catenin signaling (yellow), and angiogenesis-related processes (pink). These clusters reflect the preferential engagement of metabolic, anabolic, and differentiation-associated pathways under red and near-infrared photobiomodulation. For blue-light–responsive genes (**B**), the network displays a broader functional distribution, including apoptosis-related signaling (black), calcium signaling (gold), extracellular vesicle and exosome-associated processes (purple), inflammation and secretome-related pathways (beige), extracellular matrix organization (light blue), and oxidative stress responses. Unassigned proteins are shown in gray. Together, these network representations provide a systems-level view of how wavelength-specific photobiomodulation is associated with distinct but partially overlapping functional architectures in MSCs, supporting the concept of dual photonic programming toward regenerative versus paracrine-oriented cellular states.

High-throughput network analyses provide a systems-level framework for interpreting how red and blue PBM reorganize MSC signaling. While early studies focused on individual genes or isolated pathways, STRING-based protein–protein interaction (PPI) mapping and Gene Ontology (GO) enrichment indicate that wavelength-associated phototransduction responses distribute across distinct but partially overlapping biological networks. To integrate these literature-derived observations at the systems level, protein–protein interaction networks were constructed using STRING and visualized in Cytoscape for red-light– and blue-light–responsive gene sets (Figure 5), with Gene Ontology enrichment provided as supplementary analyses (Supplementary Figure S1).

These architectures mirror the photoreceptor-driven mechanisms described earlier: red-light responses cluster around mitochondrial metabolism, antioxidant defense, and lineage specification, whereas blue-light responses concentrate in oxidative signaling, vesicular trafficking, and paracrine communication. A comparative summary of the main functional outcomes and network-level features associated with red and blue photobiomodulation in MSCs is provided in Table 2. Together, these networks provide a mechanistic rationale for dual photonic programming of MSCs.

Beyond isolated signaling events, our systems biology analysis using STRING-based protein interaction networks (Figure 5) reveals a wavelength-dependent bifurcation in MSC behavior. The red/NIR cluster exhibits a high degree of connectivity centered on the PI3K-Akt-mTOR axis, suggesting a robust “survival and anabolic hub” that coordinates mitochondrial flux with structural differentiation. Conversely, the blue-light interactome highlights a distinct topological organization, where calcium signaling and vesicle trafficking proteins (RAB family) emerge as central nodes. This computational mapping suggests that PBM does not merely “stimulate” the cell, but executes a specific “photonic program” by engaging pre-defined protein modules. Such a systems-level perspective is essential for predicting MSC responses in complex microenvironments where multiple stimuli coexist.

### 4.1. Red-Light Network Architecture: Metabolic Reinforcement, Oxidative Resilience, and Anabolic Signaling

The STRING network generated from red-light–responsive genes shows a dense core centered on mitochondrial and redox-regulatory proteins, including CCO subunits, SOD2, HMOX1, and GPX family enzymes, and stress-response regulators such as Nrf2 pathway components [13,89]. GO enrichment supports overrepresentation of terms associated with regulated cell survival, response to oxidative stress, and redox-associated metabolic processes. These clusters are consistent with a role for red PBM in engaging mitochondrial-dependent redox signaling and bioenergetic support pathways.

Downstream nodes include PI3K/Akt/mTOR effectors, MAPKs, and Wnt/β-catenin components, capturing known links between redox modulation and commitment toward osteogenic lineages [51,72]. ECM-related terms, including collagen biosynthetic process, extracellular matrix organization, and osteoblast differentiation, emerge prominently, consistent with the enhanced ALP activity, RUNX2 induction, and mineralization reported in multiple red PBM studies [20,75,90]. Together, these network features support the interpretation that red PBM preferentially engages metabolic and anabolic signaling architectures compatible with differentiation-associated programs requiring sustained bioenergetic and biosynthetic capacity.

A secondary but biologically meaningful cluster includes anti-apoptotic and cytoprotective proteins, reflecting the ability of PBM to restore mitochondrial competence and survival under hypoxic, inflammatory, or diabetic stress [6,16]. Together, these network features depict a coherent red-light architecture in which bioenergetics, redox buffering, and differentiation signaling form a tightly integrated module positioned at the nexus of MSC fate control.

### 4.2. Blue-Light Network Architecture: Flavin-Driven Redox Microdomains, Ca^2+^ Regulation, and Secretory Programming

The STRING network generated for blue-light–responsive genes displays a markedly different topology. Rather than centering on mitochondrial nodes, the enriched clusters map to oxidoreductases, flavoproteins, circadian regulators, and vesicular trafficking components. GO terms such as response to oxidative stress, cellular response to ROS, and flavin-containing cofactor metabolic process reflect the upstream photochemistry of flavins and cryptochromes [36,83].

Pathways governing Ca^2+^ homeostasis and cytoskeletal remodeling are also enriched, consistent with blue-light–associated redox signaling events capable of influencing redox-sensitive ion channels and calcium-dependent pathways [8,18]. Importantly, genes involved in vesicular trafficking, membrane dynamics, and endosomal regulation form a distinct functional module consistent with experimental evidence reporting enhanced extracellular vesicle release and altered cargo composition under blue PBM [8,18].

Circadian and transcriptional regulators appear as an additional enriched cluster, reflecting cryptochrome involvement in blue-light photoreception and supporting observations of PBM-mediated modulation of metabolic oscillations and stress adaptation programs [80,91]. Altogether, the blue-light network architecture emphasizes paracrine activation, communication, and adaptive redox signaling, in contrast to the anabolic and differentiation-driven architecture characteristic of red-light PBM.

### 4.3. Convergent and Divergent GO-Enriched Functions Across Wavelengths

Comparative STRING/GO analysis highlights both wavelength-specific and wavelength-independent components of MSC photobiology. Both red and blue PBM enrich GO terms related to response to oxidative stress and regulation of cell proliferation, reflecting a shared dependence on ROS as signaling intermediates [78,83,89]. However, the *source*, *compartmentalization*, and *magnitude* of ROS differ dramatically between wavelengths, leading to divergent pathway activation.

Red-light networks preferentially enrich metabolic and tissue-building processes, including redox-regulated survival pathways, ossification-related functions, and extracellular matrix organization.

Blue-light networks, by contrast, enrich processes related to membrane dynamics, vesicular trafficking, calcium signaling, circadian regulation, and immune modulation [72,83]. This division reflects two preferential biological strategies engaged by distinct wavelengths: (i) red PBM optimizes the intracellular machinery required for structural regeneration; (ii) blue PBM enhances intercellular communication and stress adaptation through EV-mediated signaling.

Notably, some pathways converge, such as NRF2 activation or moderate MAPK signaling, but their modulatory context remains wavelength-specific, with red PBM favoring mitochondrial-associated redox signaling and blue PBM engaging more spatially distributed cytosolic redox responses.

Functional enrichment analysis was used as a complementary integrative tool to visualize biological processes associated with wavelength-sensitive gene sets. While STRING networks highlight distinct signaling architectures, GO enrichment (Supplementary Figure S1) supports preferential association of red photobiomodulation with regulated survival and ossification processes, and blue photobiomodulation with stress-adaptive and secretory programs.

Although transcriptomic and GO-enrichment approaches have been applied to photobiomodulation in other cell types, including dermal fibroblasts and neural tissues, comparable analyses in stem cells remain scarce [9,92,93,94]. Existing PBM-omics studies in stem cell–derived systems primarily focus on pathway-level changes without systematically contrasting wavelength-specific network architectures. In mesenchymal stem cells, high-throughput transcriptomic and GO analyses have been extensively used to characterize redox metabolism, differentiation programs, and senescence, but these datasets were generated in the absence of photobiomodulation. Recent work showing that blue and green light reshape the secretory and immunomodulatory profiles of human umbilical cord–derived MSCs further underscores the need for integrative analyses that link wavelength, transcriptional programs, and paracrine function [9]. Accordingly, the present STRING/GO comparison of red- and blue-light responses in MSCs addresses a current gap by providing a wavelength-resolved view of MSC photobiology that is not yet available in the existing literature.

A significant hurdle in the clinical translation of MSC-PBM therapies is the persistent lack of dosimetric reproducibility. Our review underscores a critical, yet frequently ignored, physical reality: the discrepancy between nominal and effective irradiance. As established by the seminal work of Hadis et al. [26], the culture architecture, specifically phenol red absorption and polystyrene refraction, acts as a non-linear filter that attenuates photon delivery to the cellular monolayer.

This energy loss, if uncompensated, inadvertently shifts the treatment from a stimulatory window into an ineffective or even inhibitory zone of the Arndt–Schultz curve. We propose that future advanced research in this field must adopt mandatory radiometric reporting standards, moving away from lamp output toward cell-delivered dose to ensure therapeutic predictability.

### 4.4. Conceptual Integration: A Dual Photonic Programming Model

Together, these network architectures support a dual-mode framework in which red and blue photons encode distinct biological information into MSC signaling. Red PBM activates a mitochondrial-centric anabolic program optimized for differentiation, matrix production, and tissue integration. Blue PBM activates a paracrine-oriented program optimized for vesicle-mediated communication, stress tolerance, and angiogenic support [72,73,95].

This dual photonic programming is not merely a spectral artifact but reflects the intrinsic alignment between wavelength-dependent photochemistry and MSC biology: (i) red/NIR wavelengths interface with mitochondrial checkpoints that dictate MSC fate; (ii) blue wavelengths interface with cytosolic redox sensors, Ca^2+^ dynamics, and secretory pathways central to MSC paracrine function. The integration of STRING-PPI modules and GO functional clusters thus provides a mechanistic systems-level understanding of how PBM can be tuned, by wavelength, dose, and temporal pattern, to produce targeted regenerative outcomes [6,13,18,83].

## 5. Photonic Programming of MSCs for Regenerative Medicine

The wavelength-dependent signaling programs identified in this review have direct implications for regenerative medicine. Red and blue PBM do not merely modulate MSC activity; they bias MSCs toward distinct therapeutic phenotypes, one oriented toward structural regeneration, the other toward paracrine-mediated repair. These complementary modes align with the dual nature of MSC function in vivo and offer a mechanistically grounded framework for designing wavelength-tailored cell therapies, light-responsive scaffolds, and MSC-derived extracellular vesicle (EV) treatments [67,72,83].

### 5.1. Red-Light Programming for Bone and Cartilage Regeneration

Among all MSC applications, skeletal regeneration exhibits the strongest and most consistent response to red and near-infrared PBM. The alignment of red-light phototransduction with mitochondrial bioenergetics directly supports the metabolic and transcriptional requirements of osteogenesis, which relies on enhanced oxidative phosphorylation, mitochondrial expansion, and ECM synthesis. Multiple studies demonstrate that red PBM increases proliferation, ALP activity, collagen deposition, mineralization, and expression of osteogenic transcription factors, including RUNX2 and Osterix [9,72,73].

The mechanistic basis lies in the mitochondria-driven activation of PI3K/Akt/mTOR and Wnt/β-catenin pathways, both essential for osteoblast lineage commitment and bone matrix assembly. Mitochondrial ROS, particularly H_2_O_2_ generated by CCO-mediated photostimulation, act as low-amplitude second messengers that stabilize β-catenin and facilitate osteo-anabolic transcriptional programs [7,13,72,89].

Red PBM has demonstrated efficacy not only in standard osteogenesis but also in enhancing the regenerative activity of periodontal ligament stem cells, adipose-derived stem cells, and meniscus-derived stem cells [75,76,96]. These effects translate into improved healing in models of bone defects, periodontal bone loss, and meniscal injury. A recent systematic review underscores red PBM as a reproducible enhancer of bone healing in both in vitro and in vivo settings [23].

In preclinical animal models, red PBM has consistently enhanced bone regeneration outcomes when MSCs are used as therapeutic agents. In rodent calvarial defect models, red or near-infrared irradiation (630–810 nm) of transplanted bone marrow MSCs significantly increased bone volume fraction, mineralization density, and vascularization at 4–8 weeks post-implantation compared with non-irradiated controls [23,88]. In alveolar bone loss models induced by ligature or extraction, periodontal ligament stem cells irradiated with red PBM exhibited improved engraftment, accelerated alveolar bone fill, and more organized periodontal fiber architecture [75,90]. In a meniscal repair model, MSC sheets preconditioned with red PBM showed superior integration and collagen fiber alignment compared with non-irradiated counterparts [96,97]. These converging translational data reinforce the in vitro mechanistic evidence described above and support the use of red PBM as an adjuvant strategy to improve MSC-based skeletal tissue engineering outcomes in vivo.

Cartilage engineering also benefits from red PBM, though via a slightly different signaling landscape. While chondrogenesis is less tightly coupled to oxidative metabolism than osteogenesis, red PBM increases SOX9 transcriptional activity and ECM gene expression under specific dosimetric conditions [24,25]. Nonetheless, the red-light–driven anabolic phenotype remains particularly suited for skeletal tissues requiring high metabolic output and matrix generation.

Taken together, these data position red PBM as a precision activator of osteogenic differentiation and skeletal tissue formation, with a mechanistic foundation robust enough to support translation into light-responsive biomaterials and scaffold-guided regeneration strategies.

### 5.2. Paracrine and Vascular Repair Enhanced by Blue-Light Programming

In contrast to red PBM, which prioritizes structural and metabolic reinforcement, blue PBM enhances MSC paracrine potency, angiogenic signaling, and immunomodulatory activity. These effects emerge from flavin-mediated ROS microdomains, cryptochrome-dependent transcriptional responses, and redox-regulated Ca^2+^ transients that stimulate vesicular trafficking [9,36,78,83].

A key discovery is that blue PBM increases the quantity and therapeutic quality of MSC-derived extracellular vesicles (EVs). Several studies report enhanced EV biogenesis, altered miRNA cargo composition, and improved EV-driven angiogenesis and wound healing following blue-LED exposure [18,21]. The EVs generated under blue PBM stimulate endothelial migration, promote microvascular formation, and modulate immune responses, effects attributed to ROS- and Ca^2+^-dependent remodeling of the ESCRT machinery and membrane curvature dynamics [9,83,98].

This wavelength-dependent strategy of producing wavelength-programmed EV therapeutics represents a promising translational direction in PBM research, enabling the generation of EVs specialized for metabolic rescue (red), EVs optimized for angiogenesis and immune modulation (blue), and potentially sequential or mixed EV formulations. This paradigm aligns with recent developments in EV engineering and advanced drug delivery, positioning PBM as a scalable and controllable approach for modulating EV production in regenerative medicine [67,83,97].

Although direct in vivo evidence for blue-light PBM specifically in MSC transplantation models remains lacking, in vitro data from [8,18] support the concept that blue PBM amplifies MSC paracrine potency.

### 5.3. Sequential or Combined Photonic Programming: An Emerging Strategy

**Figure 6:**
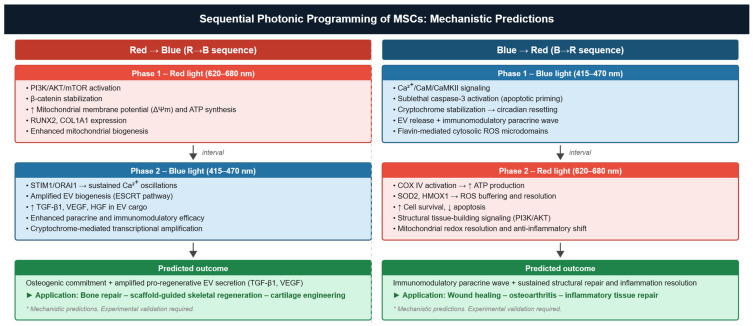
Conceptual framework for sequential photonic programming of mesenchymal stem cells. Predicted mechanistic cascades for R-B (**left panel**) and B–R (**right panel**) sequential illumination protocols. R–B: red-light preconditioning stabilizes mitochondrial bioenergetics and PI3K/AKT/mTOR signaling before blue-light exposure amplifies STIM1/ORAI1–dependent Ca^2+^ oscillations and extracellular vesicle biogenesis, with predicted pro-regenerative paracrine output. B–R: blue–light preconditioning engages CaMKII/caspase–3/cryptochrome pathways and EV release before red–light redirects mitochondrial output toward cytoprotection and anti–inflammatory resolution. These mechanistic predictions require systematic experimental validation.

Although red and blue PBM have been extensively characterized separately, only limited studies have explored their combined or wavelength-combination effects in MSC modulation. Existing reports in other biological systems suggest that distinct spectral bands can produce additive or synergistic effects, but these protocols have not been mechanistically dissected in MSCs. The strong mechanistic divergence uncovered in this review, with orthogonal photoreceptors, non-overlapping ROS compartments, and distinct downstream signaling programs, nevertheless, provides a rational, hypothesis-generating basis for exploring multi-wavelength strategies. The two scenarios presented below (Figure 6) are explicitly formulated as mechanistic hypotheses. They are not supported by direct experimental evidence in MSCs and require systematic validation before any translational inference can be drawn.

**Hypothesis** **1.** *Red-to-blue (R to B) sequential programming*.

*Experimental Basis:* Red/NIR PBM reproducibly enhances mitochondrial membrane potential, ATP production, and PI3K/Akt/mTOR and Wnt/beta-catenin signaling in MSCs [7,72]. Blue PBM independently increases cytosolic Ca^2+^ transients, EV biogenesis, and paracrine potency through flavin-mediated ROS microdomains [18,21,83].

The metabolically reinforced state created by prior red PBM exposure is assumed to sensitize MSCs to blue-light–driven Ca^2+^ signaling by enhancing STIM1/ORAI1 responsiveness and optimizing mitochondrial network fusion. Neither of these interactions has been demonstrated experimentally in a sequential context.

We hypothesize that preconditioning with red light (620–680 nm) followed by blue-light exposure (415–470 nm) would yield amplified EV biogenesis with enhanced pro-angiogenic miRNA cargo, compared with either wavelength applied alone. This prediction could be tested by applying the two wavelengths sequentially to AD-MSCs under standardized dosimetric conditions and quantifying EV output, cargo composition (miR-21, miR-126), and endothelial tube formation as functional readouts.

**Hypothesis** **2.** *Blue-to-red (B to R) sequential programming*.

*Experimental Basis:* Blue PBM engages cytosolic ROS microdomains, Ca^2+^/CaM/CaMKII signaling, and cryptochrome-dependent transcriptional reprogramming in MSCs [81,82,83]. Subsequent red/NIR PBM enhances mitochondrial ATP production and anti-apoptotic signaling [13,15,16].

CRY2-mediated suppression of RUNX2 and the secretory state induced by blue PBM are assumed to create a permissive microenvironment in which subsequent red-light metabolic reinforcement redirects energy output toward anti-inflammatory resolution rather than osteogenic differentiation. This assumption rests on the sequential interaction of two independently characterized pathways and has not been tested in any combined protocol.

We hypothesize that a B-to-R sequence would be particularly suited to inflammatory microenvironments, first enhancing immunomodulatory EV release and then reinforcing MSC survival under inflammatory stress. This could be evaluated in co-culture or conditioned-medium models using lipopolysaccharide-activated macrophages, measuring TNF-alpha and IL-10 secretion, MSC viability, and mitochondrial respiration before and after each illumination phase.

These hypotheses remain speculative and require systematic experimental validation. No conclusion regarding clinical application should be drawn from these mechanistic predictions in the absence of direct evidence in MSCs.

## 6. Comparative Photobiomodulation Responses Across Mesenchymal Stem Cell Sources

Although the mechanistic framework described in this review draws primarily on evidence from adipose-derived MSCs (AD-MSCs) and bone marrow–derived MSCs (BM-MSCs), comparable wavelength-specific responses have been documented across multiple tissue-specific progenitor populations, underscoring the generalizability of the dual photonic programming concept.

Adipose-derived MSCs (AD-MSCs) hold a central position in this comparative analysis as the primary cell source from which the dual photonic programming framework is derived. Red PBM drives robust osteogenic and matrix-producing programs in AD-MSCs through PI3K/Akt/mTOR and Wnt/β-catenin pathway activation [7,72], with functional effects extending to enhanced differentiation into tenocyte and neural lineages [7,21]. Strikingly, AD-MSCs appear particularly sensitive to blue PBM in terms of paracrine amplification: blue irradiation enhances extracellular vesicle biogenesis, angiogenic cargo loading, and immunomodulatory secretory output at relatively low fluences [18,83]. This pronounced blue-light responsiveness may reflect the high cytosolic flavin content of adipose-derived progenitors and their intrinsically elevated paracrine identity, positioning AD-MSCs as efficient transducers of blue photons into secretory and angiogenic signals. These properties make AD-MSCs a compelling model for dual photonic programming and a clinically relevant candidate for PBM-enhanced therapies in wound healing, vascular repair, and soft-tissue regeneration.

BM-MSCs exhibit robust osteogenic and cytoprotective responses to red and near-infrared PBM. Studies in normal and osteoporotic BM-MSCs demonstrate that 650–810 nm irradiation increases alkaline phosphatase activity, matrix mineralization, and PI3K/Akt/mTOR signaling, with particularly pronounced effects in metabolically impaired or aged cells [65,66,68]. Notably, PBM has been shown to rejuvenate aged BM-MSCs by restoring mitochondrial membrane potential and redox homeostasis [6], a function directly relevant to clinical settings where donor-age–related MSC decline limits therapeutic efficacy.

Dental pulp stem cells (DPSCs), shaped by their neural crest developmental origins, present a distinct photobiology profile. Blue light enhances DPSC osteogenic differentiation through transient receptor potential channel (TRPC)-mediated calcium influx [85], while combined blue and near-infrared irradiation acts synergistically on both metabolic and calcium-signaling axes [99]. These findings suggest that wavelength-combination strategies may be uniquely effective in DPSCs, potentially leveraging their neurogenic and secretory repertoire for craniofacial regeneration.

Periodontal ligament stem cells (PDLSCs), a craniofacial-specific progenitor population, show clear red PBM sensitivity: 660–980 nm irradiation enhances osteoblastic differentiation, bone-like tissue formation, and matrix organization in vitro and in alveolar bone loss models [75,90]. Their responsiveness mirrors that of BM-MSCs and AD-MSCs along the osteogenic axis, suggesting that CCO-mediated mitochondrial activation is a conserved mechanism across connective tissue progenitors regardless of tissue of origin.

Human umbilical cord–derived MSCs (hUCMSCs), valued for their high paracrine activity and low immunogenicity, respond to blue and green PBM with enhanced proliferation, improved wound healing support, and increased secretory output [9]. The order of wavelength application influences neural differentiation trajectories in cord matrix–derived MSCs [100], aligning with the sequential photonic programming concept and suggesting that temporal light control may be broadly applicable across MSC sources.

Across sources, a conserved architecture emerges: red/NIR PBM consistently enhances metabolic fitness, survival signaling, and structural differentiation, while blue PBM preferentially modulates secretory and adaptive programs. Source-specific differences are most apparent in the magnitude of differentiation responses and in blue-light oxidative threshold sensitivity, likely reflecting variations in chromophore abundance, mitochondrial density, and baseline redox tone. These observations motivate head-to-head comparative studies under standardized dosimetric conditions to establish wavelength–dosimetry windows tailored to each clinically relevant MSC population (Table 3).

## 7. Conclusions

Photobiomodulation exerts wavelength-specific control over mesenchymal stem cell behavior through distinct photophysical and biochemical mechanisms. Red and near-infrared light engage cytochrome c oxidase–associated redox signaling, generate controlled mitochondrial hydrogen peroxide signals, and activate PI3K/Akt, ERK, and Wnt/β-catenin pathways, thereby supporting survival, metabolic reinforcement, and differentiation-compatible signaling states. In contrast, blue light triggers flavin-mediated photochemistry, cryptochrome-dependent transcriptional programs, localized cytosolic redox signaling, and calcium remodeling, resulting in enhanced paracrine communication and extracellular-vesicle–associated signaling.

Consistent with experimental evidence from the literature, STRING and Gene Ontology analyses support the notion that these photonic modes propagate through divergent intracellular network architectures, with red photobiomodulation preferentially enriching mitochondrial- and anabolic-associated modules, and blue photobiomodulation emphasizing vesicular trafficking, circadian regulation, and adaptive oxidative signaling processes.

Together, these findings establish that photobiomodulation is not a uniform biophysical stimulus, but rather a dual photonic programming system capable of biasing MSC functional states toward structurally regenerative or communication-oriented phenotypes. This mechanistic framework provides a rational basis for wavelength-tailored therapeutic strategies, including the use of red photobiomodulation to support skeletal regeneration and metabolic rescue, and blue photobiomodulation to potentiate vascular repair, immunomodulation, and extracellular-vesicle–based approaches. The complementary nature of these pathways further suggests that sequential or combined multispectral photobiomodulation, although not yet experimentally validated, represents a promising frontier for engineering multi-phase regenerative responses.

Future studies should integrate wavelength-specific mechanisms with optimized dosimetry, temporal control, and biomaterial-based light delivery, particularly in physiologically relevant and pathological microenvironments. By aligning photonic inputs with MSC metabolic, redox, and secretory architecture, photobiomodulation emerges as a powerful, non-invasive strategy for precision control of stem cell function and a scalable platform for next-generation regenerative medicine.

## Figures and Tables

**Table 1:** Overview of photobiomodulation parameters reported in stem cell studies.

Application	Light Source Parameters						Therapeutic Parameters			
	Wavelength (nm)	Power (mW)	Power Density (mW/cm^2^)	Each Treatment Duration (s)	Energy Density (J/cm^2^)	CW or Pulsed Laser	Source of Stem Cells	Distance from Tissue (mm)	Timing per Session (s)	Number of Sessions
**Diabetic ulcer**	630, 810, 890	0.05–1.08	0.001–1.08	46–92	0.2–2.4	pulsed	BM-MSCs, ADSCs		200	15–16
**Wound healing**	532, 630–660, 808–890	17–100	0.625–100	4–600	0.1–55.36	CW	ADSCs, BMSCs, DPSCs, MSCs, ESCs	1.2–100	20–152	13–20
**Proliferation and viability**	630–660, 800–830	10–313	0.33–156	3–300	0.5–180	CW or pulsed	DPSCs, SHED, MSCs, ADSCs, BM-MSCs, hRPESCs, WJMSCs, PDLSCs, TSCs, OEC, ORSCs	5–150	10	2–5
**Angiogenesis**	475, 516, 630–660, 800–830	400	1.65–80	20–150	0.3–30		ADSCs, HUVECs, MSCs, HUGBSCs, BMSCs	20–80	10–600	20
**Bone regeneration**	405, 525, 630–660, 780–910	3–500	0.2–714	20–46	0.4–13.3	CW or pulsed	MSCs, BMSCs, ADSCs	5–66	20	21
**Muscle regeneration**	630–685, 780–980	4.5–17.6	0.25–440	3–20	0.08–5		Myoblast SCs			
**Nerve regeneration**	630–660 and 808	200–750	20–100	1	0.05–9	CW	MSCs, NSCs			7
**Tendon regeneration**	532, 660, 808, 905	40		120	5–50	CW or pulsed	TDSCs, MSCs, ADSCs	2	70	
**Cartilage regeneration**	808–810	30–50	430	20	8.5–71.2		MSCs, ADSCs	5		
**Cancer**	470, 630–660, 835–850	10–100	1.07–110		3–500	CW	ADSCs, HMSCs, LCSCs, CSCs, PHSCs	150		
**Tooth**	660, 780, 940, 980	20–200	1–714	10–80	1–42	CW	MSCs, DPSCs, SHED	15–20		
**Osteoporosis**	633, 810	3–50	1.6–26	378	0.6–2.4	CW	BM-MSCs	100–150		
**Heart**	635, 804–808	5–900	6.37–10	100–150	0.96–1	CW	BM-MSCs			
**Differenciation bone**	420, 485, 532–540, 630–660, 805–810, 940, 1064	1.5–1000	0.5–1333	3–600	0.5–90	CW or pulsed	MSCs, BMSCs, ADSCs, HUMSCs, DPSCs, PDLSCs	5–200		3–56
**Differentiation gametogenic**	625		5.3		1.9		hWJMSCs			
**Differentiation muscle**	630–660	60–85	6.61–9.3	75–750	0.5–5	CW	BM-MSCs, ADSCs			
**Differentiation Chondrocyte**	630–660	97.1–98.25	10.6–10.8	7.42–7.48	5	CW	ADSCs, MSCs			
**Differentiation neural**	525, 980, 630–660, 808–825	30–574	11.45–167	18–36	3–15	CW	BM-MSCs, hUC-MSCs, DPSCs, ADSCs, Impacted tooth MSCs			
**Differentiation Osteoclast**	810	50			9.33–93.30		Rat osteoclast precurser cell			
**Differentiation Inner ear chair**	630	40		750	30	CW	Rat embryonic SC			
**Differentiation Adipogenic**	1064				8.8–26.4		hMSCs			
**Alzheimer’s disease**	808	400		20	1		NSCs			6
**Inflammatory Reaction**	660	3–70	3–15.17	264–528	4–16		ADSCs, MSCs			
**Cell sheets**	660	10–30	710	4–10	2.5–7.5	CW	hDPSCs, SHED			
**CFU**	600, 685, 850	25–190	3–10	38–770	0.1–3	CW				
**COPD**	660	30		180						
**Oxidative stress**	633	1.15	1.15	378	1.5	Pulsed	BM-MSCs			
**Pain**	660	100		45		CW	ADSCs	5		14
**Kidney**	405, 532, 635, 804	18–400	10–12.2	100–300	0.036–1	CW	MSCs, BM-MSCs			

Summary of light-source characteristics and therapeutic parameters used in published photobiomodulation studies involving stem cells, including wavelength, power, energy density, irradiation mode (continuous wave or pulsed), biological application, cell source, irradiation geometry, and treatment regimen. Data were compiled from 204 peer-reviewed research articles identified through a comprehensive literature search of PubMed, Web of Science, and Scopus using the terms “photobiomodulation”, “low-level laser therapy”, “LLLT”, “LED therapy”, and “stem cells”, spanning three decades of the published literature. Dosimetric reference ranges were taken from WALT dosimetry recommendations (World Association for Photobiomodulation Therapy; https://waltza.co.za, Accessed on 23 January 2026). CW: continuous wave. Table adapted from reference [41]. BM-MSC: bone marrow–derived mesenchymal stem cell; AD-MSC: adipose-derived mesenchymal stem cell; DPSC: dental pulp stem cell; SHED: stem cells from human exfoliated deciduous teeth; hUCMSC: human umbilical cord mesenchymal stem cell; SC: stem cell; NSC: neural stem cell.

**Table 2:** Wavelength-dependent bifurcation of intracellular signaling in MSCs. The dual-color coding scheme represents the distinct signaling trajectories activated by photobiomodulation. The red/pink-shaded area (left) denotes the Anabolic/Mitochondrial program triggered by red/NIR light (600–660 nm), focusing on cytochrome c oxidase activation and bioenergetic reinforcement. The blue-shaded area (right) denotes the Secretory/Paracrine program induced by blue light (400–500 nm), which primarily engages flavin-mediated ROS microdomains and calcium signaling to modulate the MSC secretome. This visual separation underscores that different wavelengths execute qualitatively distinct biological programs rather than mere variations in stimulatory magnitude.

	Red Light	Blue Light
**String Network**	Survival, differentiation and angiogenesis-centered; hubs: HMOX1, SOD2, RUNX2, AKT1, VEGFA	Stress, secretion and apoptosis-related clusters; hubs: SOD2, GPX1, BAX, MMPs
**Top Go Processes**	Regulation of cell death, ossification, oxidative stress response, apoptotic regulation	Apoptotic signaling, oxidative stress, increases secretome

Comparative summary of network-level features and functional outcomes associated with red and blue photobiomodulation in mesenchymal stem cells.

**Table 3:** Conceptual summary of how red and blue photobiomodulation may differentially and complementarily contribute to regenerative applications, based on published experimental studies.

Application	Role of Red Light	Role of Blue Light	Combined Benefit	Ref.
**Tissue regeneration**	Reported to enhance osteogenic differentiation and ECM production; Enhances angiogenesis in vivo	Modulates oxidative stress, paracrine activity	Denser tissue, improved integration	[12,92]
**Cell-free therapies (exosomes)**	Enhances viability and differentiation of MSCs	Reported to increase extracellular vesicle release	Increased production of regenerative factors	[11,86]

## Data Availability

No new data were created.

## Supplementary Materials

The following supporting information can be downloaded at: https://www.mdpi.com/article/10.3390/cells15100861/s1, Figure S1: Gene Ontology (GO) biological process enrichment of red-light– and blue-light–responsive gene sets in mesenchymal stem cells. Table S1: The complete protein lists studied for each wavelength condition.

## Abbreviations

The following abbreviations are used in this manuscript:

MSC	Mesenchymal stem cell
PBM	Photobiomodulation
NIR	Near-infrared
CCO	Cytochrome c oxidase
ROS	Reactive oxygen species
mtROS	Mitochondrial reactive oxygen species
EV	Extracellular vesicle
GO	Gene Ontology
PPI	Protein–protein interaction
CRY	Cryptochrome
FAD	Flavin adenine dinucleotide
FMN	Flavin mononucleotide
CaMKII	Ca^2+^/calmodulin-dependent protein kinase II
PI3K	Phosphoinositide 3-kinase
mTOR	Mechanistic target of rapamycin
ALP	Alkaline phosphatase
RUNX2	Runt-related transcription factor 2
VEGF	Vascular endothelial growth factor
HGF	Hepatocyte growth factor
LED	Light-emitting diode
WALT	World Association for Photobiomodulation Therapy
BM-MSC	Bone marrow–derived mesenchymal stem cell
AD-MSC	Adipose-derived mesenchymal stem cell
DPSC	Dental pulp stem cell
ESCRT	Endosomal sorting complexes required for transport
