# Supplementary material for: Wavelength-Dependent Modulation of Mesenchymal Stem Cell Fate: A Systems Biology Framework for Tissue Repair and Regenerative Medicine
Source: Cells. 2026 May 8;15(10):861. doi: 10.3390/cells15100861 (PMC13204864; doi:10.3390/cells15100861)
Supplement: Supplementary file 1 [file cells-15-00861-s001.zip › Supplementary_Table1_titled.pdf]

Supplementary Table 1. Published photobiomodulation studies in MSCs: detailed parameter summary and biological outcomes.

	Red Light Protein analysis	Blue Light Proteins analysis
1	HMOX1	CACNA1C
2	HSPA1A	CACNA1H
3	HSP90AA1	CACNA1A
4	SOD2	ATP2B1
5	GPX1	ATP2A2
6	RUNX2	SLC8A1
7	BGLAP	ORAI1
8	SPP1	STIM1
9	IBSP	RYR1
10	TGFB3	ITPR1
11	SOX9	CALM1
12	COL2A1	CAMK2A
13	COL1A1	CAMK2B
14	VEGFA	PPP3CA
15	FGF2	RUNX2
16	MMP1	SP7
17	MMP2	DLX5
18	MMP3	COL1A1
19	MMP9	BGLAP
20	TIMP1	IBSP
21	PIK3CA	SPP1
22	PIK3CB	BMP2
23	PIK3CD	BMP4
24	PIK3CG	TGFB1
25	AKT1	WNT3A
26	AKT2	CTNNB1
27	AKT3	TSG101
28	MTOR	PDCD6IP
29	RICTOR	SMPD3
30	RPTOR	VPS4B
31	CTNNB1	CHMP4B
32	APC	RAB27A
33	AXIN1	RAB11A
34	GSK3B	RAB7A
35	LEF1	RAB5A

36	TCF7L2	CD9
37		CD63
38		CD81
39		IL6
40		CXCL8
41		VEGFA
42		TGFB1
43		SOD1
44		SOD2
45		GPX1
46		GPX4
47		CAT
48		PRDX1
49		NFE2L2
50		KEAP1
51		HMOX1
52		UCP2
53		CYCS
54		NOX4
55		NQO1
56		CASP3
57		CASP7
