# Supplementary figures and images for: Wavelength-Dependent Modulation of Mesenchymal Stem Cell Fate: A Systems Biology Framework for Tissue Repair and Regenerative Medicine
Source: Cells. 2026 May 8;15(10):861. doi: 10.3390/cells15100861 (PMC13204864; doi:10.3390/cells15100861)

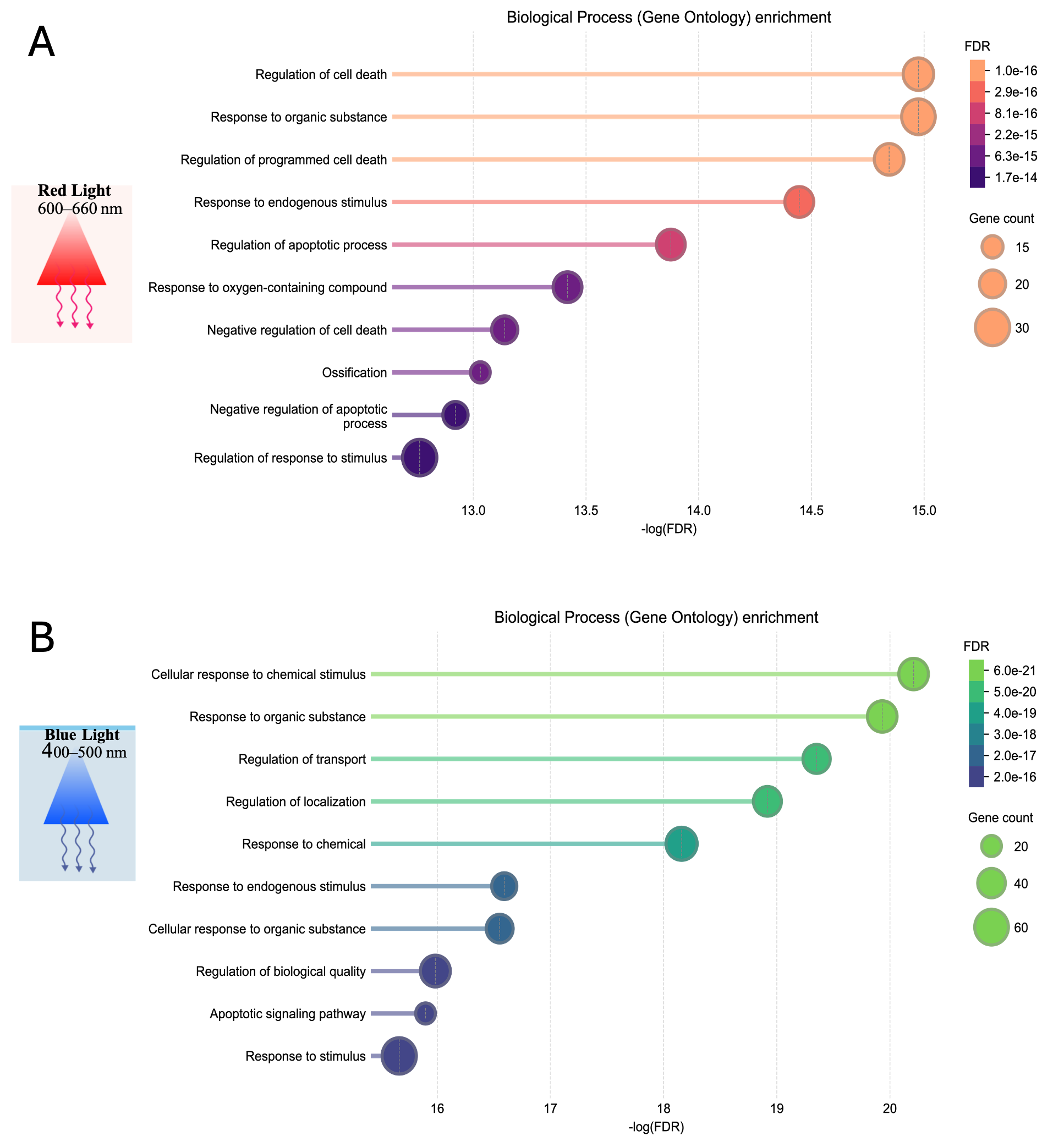

Supplement: Supplementary file 1 [file cells-15-00861-s001.zip › Figure S1.png]
